# A Critical Realist Translational Social Epidemiology Protocol for Concretising and Contextualising a “Theory of Neighbourhood Context, Stress, Depression, and the Developmental Origins of Health and Disease (DOHaD)”, Sydney Australia

**DOI:** 10.5334/ijic.3962

**Published:** 2019-07-25

**Authors:** John G. Eastwood, Lynn A. Kemp, Pankaj Garg, Ingrid Tyler, Denise E. De Souza

**Affiliations:** 1 School of Women’s and Children’s Health, The University of New South Wales, Sydney, NSW, AU; 2 Ingham Institute of Applied Medical Research, Liverpool, NSW, AU; 3 Charles Perkins Centre, Menzies Centre for Health Policy, Discipline of Child and Adolescent Health, School of Public Health, University of Sydney, Sydney, NSW, AU; 4 Sydney Institute for Women, Children and their Families, Sydney, NSW, AU; 5 Department of Community Paediatrics, Croydon Community Health Centre, Sydney Local Health District, Croydon, NSW, AU; 6 Translational Research and Social Innovation (TReSI), School of Nursing and Midwifery, Western Sydney University, Penrith, NSW, AU; 7 Dana Lana School of Public Health, University of Toronto, Toronto, ON, CA; 8 Fraser Health Authority, Surrey, BC, CA; 9 School of Humanities and Social Sciences, Nanyang Technological University, SG; 10 Discipline of Child and Adolescent Health, School of Medicine, University of Sydney, Sydney, NSW, AU

**Keywords:** critical realism, evaluation, theory, developmental origins of health and disease, neighbourhood, social epidemiology, translational epidemiology

## Abstract

**Background:**

We will describe here a translational social epidemiology protocol for confirming a critical realist “Theory of Neighbourhood Context, Stress, Depression, and the Developmental Origins of Health and Disease (DOHaD). The approach will include the concretising and contextualising of the above causal theory into programme theories for child and adolescent interventions that aim to break intergenerational cycles of disadvantage and poor life outcomes. In undertaking this work we seek to advance realist translational methodology within the discipline of applied perinatal and paediatric social epidemiology.

**Theory and Methods:**

The research settings are in metropolitan Sydney. The design will be a longitudinal, multi-level, mixed method realist evaluation of applied programme interventions that seek to break the intergeneration cycle of social disadvantage and poor child health and developmental outcomes. The programme of research will consist of three components: 1) **Operationalisation** of the theory and designing of programme initiatives for implementation; 2) **Evaluation** of the translated programme and implementation theory using Theory of Change and critical realist evaluation; and 3) **Theory Testing** of realist hypotheses using both intensive and extensive critical realist research methods including realist structural modelling.

**Discussion:**

The proposed programme of research will assist in translating empirical explanatory theory building to theory driven interventions. The research will be situated in socially disadvantaged regions of Sydney where the local child and family inter-agencies will collaborate to design and implement new initiatives that address significant disparities in childhood development and adolescent outcomes attributed to neighbourhood circumstances, family stress and intergenerational cycles of disadvantage and poor mental health.

## Introduction

The importance of the early years as a determinant of later chronic disease, mental illness, crime and adverse health and social outcomes, is increasingly being recognised [1,2]. Exposure to adversity during sensitive periods of development have been implicated as contributing to lasting changes in brain structure, emotional regulation and neuro-endocrine function through complex nutrition, metabolic and epigenetic mechanisms [3]. The environmental influences are proposed to be hierarchical including intrauterine, family and household life, and external physical and social environments [4,5]. This life course approach is not temporally limited to one generation but has a complex biological and social link across generations that includes a potential role for family households, culture, local neighbourhoods and regional or national influences [6].

The importance of the early years is increasingly being recognised by policy makers across governments in high income countries [7,8]. The policy response has acknowledged the requirement to intervene across government sectors and civil society utilising both evidence-informed interventions and, integrated structural and process strategies within complex public sector systems. The Australian state of New South Wales implemented an early childhood (0–8 years) and family focused interagency response, called *Families First*, in 1998 [9]. The NSW interagency response attempted to improve health, development and wellbeing outcomes through the implementation of a wide range of multi-faceted elements in health, education, local government and social care sectors. While that initiative included a number of evidence-based interventions [10], Fischer and colleagues noted that it suffered from structural and processes problems [11].

The NSW health sector interventions were informed by the disciplines of social epidemiology [12,13], early childhood [14] and the extensive research base related to nurse home-visiting [15]. The work of Starfield [16], Lynch and colleagues [17] and Muntaner [18] drew attention to the need to develop a theoretical understanding of the systems at play, and the complex interplay of context, mechanisms and observed outcomes.

In response to this challenge we undertook critical realist programme of research, in South Western Sydney, that sought to build a “Theory of Neighbourhood, Stress, Depression and the Developmental Origins of Health and Disease (DoHD)” using maternal postnatal depression as a case-study [19,20].

**Figure 1:**
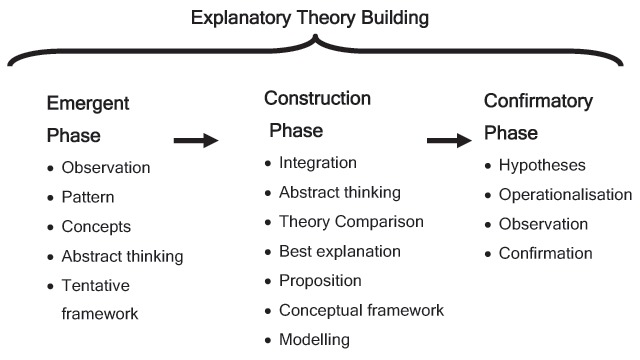
Phases of Explanatory Theory Building.

Multi-level mixed method studies were used to build a realist middle-range theory of “Neighbourhood Context, Stress, Depression, and the Developmental Origins of Health and Disease (DOHD)” [21] using an *Explanatory Theory Building Method* [19] (Figure 1). The findings of the Emergent and Construction Phases have been previously reported [22,23,24,25,26,27,28].

**Figure 2:**
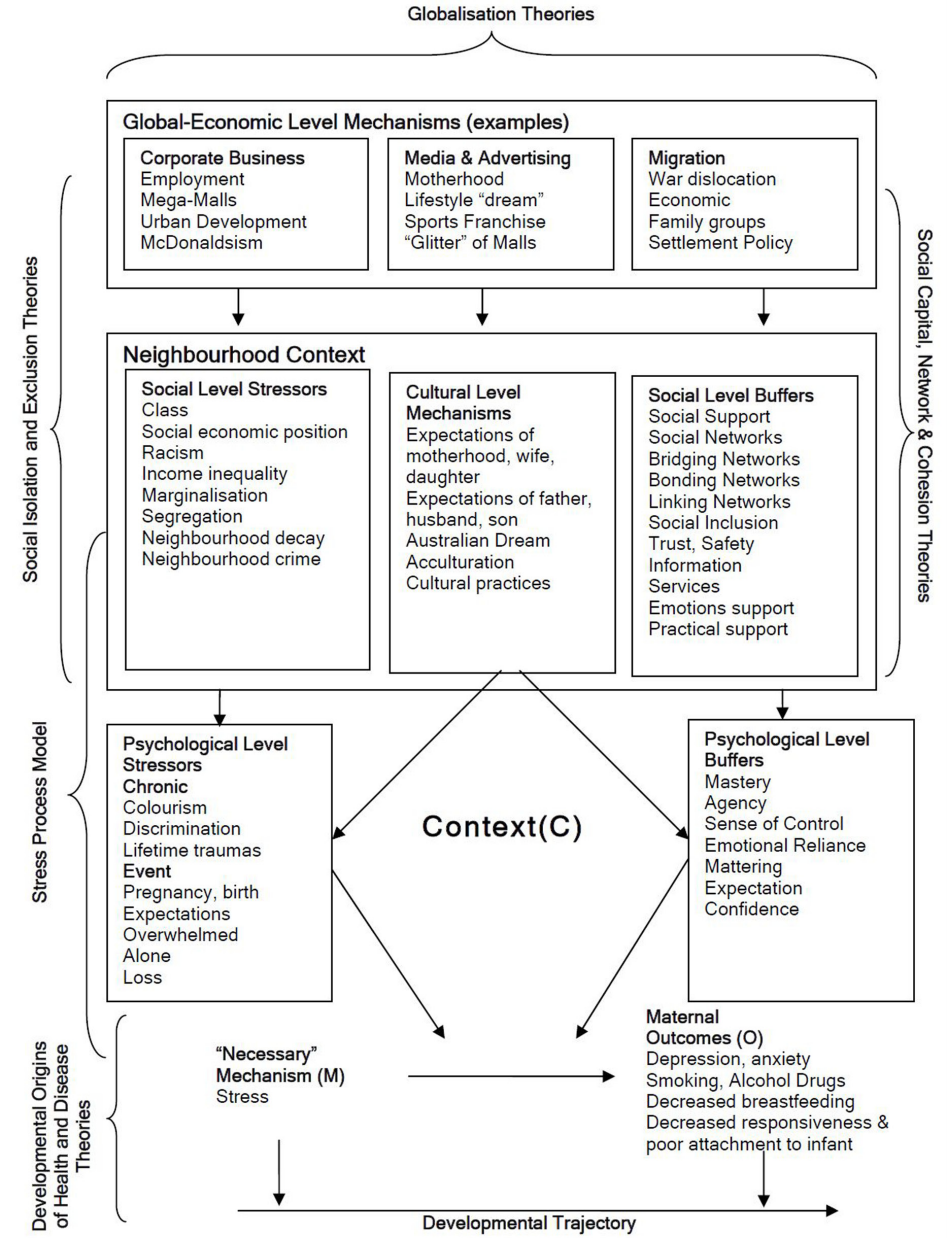
Conceptual Framework of Maternal Depression, Stress, and Context.

A conceptual framework of maternal depression, stress and context was described (Figure 2).

Muntaner [29] has subsequently argued for the use of a realist methodology that seeks to generate social interventions in partnership with the affected populations. In making this argument Muntaner was challenging social epidemiologists to move from the study of causal mechanisms (i.e. realist causal theory) toward the applied development of implementation and programme theories [30]. We seek to contribute towards this challenge, and describe a realist translational social epidemiology methodology for the translation of empirically ‘data derived’ causal middle-range theories of social mechanisms, into social programmes (with programme theories). Those theoretical propositions can be operationalised and studied in concrete situations using theory driven approaches. The programme of research will be undertaken in metropolitan Sydney, Australia.

The *Confirmatory Phase* of research seeks to confirm, further develop and test the middle-range theories constructed. We will describe here a methodology for the *Confirmatory Phase* with an emphasis on the concretising and contextualising of theory in applied programme interventions that seek to:

1. Break the intergeneration cycle of social disadvantage and poor child health and developmental outcomes.
2. Move from explaining the underlying social mechanisms to generating social interventions.

## Theory and Methods

### Critical Realism

A number of authors have elaborated the application of critical realism to both theory development and theory testing [31,32,33,34,35,36,37,38]. The purpose here is to introduce the central tenants of the critical realist meta-theory relevant to the methodology described below.

We contend as articulated by Bhaskar [39], that critical realism is an appropriate meta-theory for both the generation of causal explanations in social epidemiology and their translation to social interventions as well as confirmation of such causal theories and the evaluation of their concretisation and contextualisation of social interventions. We have previously noted that:

*“Critical realists perceive that reality consists of unobservable elements beyond our empirical realm that are still reachable by scientific inquiry. In arguing that social reality can be known, even though the social world is unpredictable and complex, critical realism offers a conception of the real that is fundamentally different from the empirical realism of the natural sciences. A central aspect of critical realism ontology is the distinction between three ontological domains: the empirical, the actual, and the real. The empirical domain comprises our experiences of what actually happens (i.e., experiences), and the actual is made of things that happened independently of whether or not we observed them (i.e., events). The last ontological domain, the real, is the deepest level of reality and is constituted by mechanisms with ‘generative power’*.

*A second critical realist ontological dimension is that reality is stratified. Reality is assumed to consist of hierarchically ordered levels, where a lower level creates the conditions for a higher level. The higher level is not, however, determined by the lower level and has its own ‘generative mechanisms’. The existence of these level-specific generative mechanisms is what constitutes or defines a level. The different levels, or strata, have been variably described as including physical, chemical, biological, psychological, psychosocial, behavioral, social, cultural, and economic components. Each stratum is separate and distinct and may interact with the layer above or below to produce new mechanisms, objects, and events”* [27].

**Figure 3:**
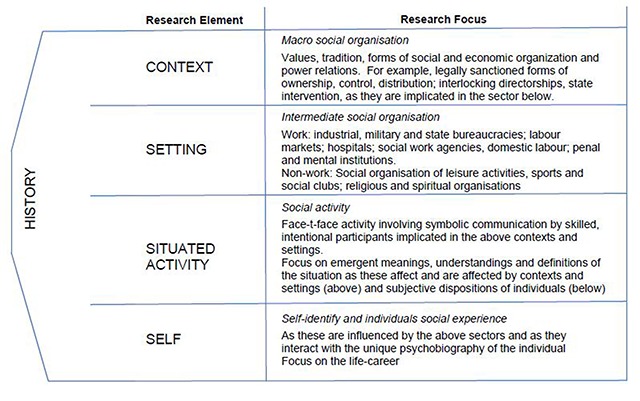
Research Map [41, p 72].

The ability of mechanisms to combine to create something new is called *emergence*. It is the existence of these level-specific mechanisms that will define an ontological level or layer within what Bhaskar and Danermark call a laminated system [40]. Layder [41] illustrated this layering of reality in his Research Map [41] (Figure 3). *Emergence* and the hierarchy of levels are both central tenants of the confirmatory and evaluation methodology described in this protocol. Important also is the analysis of pre-existing historical structural elements.

The idea that an event will not always follow from a causal mechanism, in an open system, is called a *tendency*, where the contextual conditions for the mechanisms to operate may not exist. *Context*, thus, determines how a mechanism is empirically manifest [42,43,44]. The concept of mechanisms is central to realist ontology. Such mechanisms can exist beneath the empirical surface in the *real* domain and, therefore, are not directly observable. Thus, for realists explanation depends on identifying causal, or program, mechanism and how they work, and discovering if they have been activated and under what conditions” [45, p 14].

**Figure 4:**
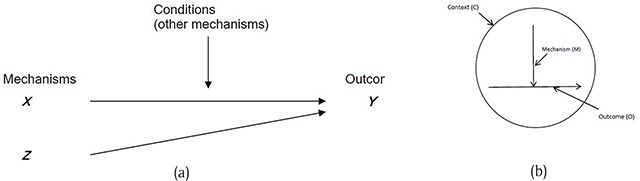
CMO Propositions: **(a)** Danermark et al [35]; **(b)** Pawson and Tilly [34].

Realist causal propositions are expressed in terms of mechanisms (M), context (C), and outcomes (O). The MCO propositions in our previously reported theory [20] are in the form proposed by Danermark and colleagues [35] (Figure 4a). For evaluation studies, Pawson and Tilley [34] propose a CMO configuration as in Figure 4b.

In realist programme evaluation terminology the mechanism (M) is an intervention mechanism (IM), and not a causal mechanism. Denyer and colleagues [46] draw attention to the importance of specifying the intervention separate from the mechanism and proposed the use of a CIMO-logic (Context, Intervention Mechanism, Outcome). Thus a CIMO is a hypothesis that the programme theory produces a change (O) because of the action of an intervention (I) on an underlying mechanism (M) operating in particular contexts (C) Table 1.

**Table 1:** CIMO-logic – the Components of Design Propositions [46].

Component	Explanation

Context (C)	The surrounding (external and internal environment) factors and the nature of the human actors that influence behavioural change. They include features such as age, experience, competency, organizational politics and power, the nature of the technical system, organizational stability, uncertainty and system interdependencies. Interventions are always embedded in a social system and, as noted by Pawson and Tilley (1997), will be affected by at least four contextual layers: the individual, the interpersonal relationships, institutional setting and the wider infrastructural system.
Interventions (I)	The interventions managers have at their disposal to influence behaviour. For example, leadership style, planning and control systems, training, performance management. It is important to note that it is necessary to examine not just the nature of the intervention but also how it is implemented. Furthermore, interventions carry with them hypotheses, which may or may not be shared. For example, ‘financial incentives will lead to higher worker motivation’.
Mechanisms (M)	The mechanism that in a certain context is triggered by the intervention. For instance, empowerment offers employees the means to contribute to some activity beyond their normal tasks or outside their normal sphere of interest, which then prompts participation and responsibility, offering the potential of long-term benefits to them and/or to their organization.
Outcome (O)	The outcome of the intervention in its various aspects, such as performance improvement, cost reduction or low error rates.

We will use CIMO here to differentiate the programme hypotheses from causal hypotheses as used by other realist methodologists [35,36].

### Explanatory Theory – Confirmatory Phase

We propose to use in this programme of research the *Confirmatory Phase* of *Explanatory Theory Building Method*, introduced above (Figure 1) [19]. *Explanatory Theory Building Method* uses induction, abduction, retroduction and deduction as the central forms of reasoning moving from description of the concrete, to the abstract, and back to the concrete [35, p 109–111].

In this approach the researcher begins with descriptive and exploratory examination of the phenomena, events and situations intended for study. This is followed by an analytical process that involves identification of components, abduction and retroduction, comparison of theories and abstractions, and concretisation studies of the theorised mechanisms in different (contextual) situations. The *Confirmatory Phase* builds on the Concretisation and Contextualisation stage described by Danermark and colleagues [35]. Realist hypotheses are developed from the theoretical propositions, operationalised, and studied in concrete situations. We summarise below two approaches as elaborated by: Sayer [36,45] and Pawson and Tilley [34].

Realist research methods proposed by Sayer [36,45] can be used for both the development and confirmation of realist causal hypotheses. Sayer [36] emphasised the importance of different methods of data collection and analysis. He proposed four types of research: intensive or concrete (empirical and theoretical analysis); generalisation (empirical), abstract (theoretical) and synthesis (interdisciplinary analysis). Sayer [45] further outlines two different kinds of research design relevant to the programme of research described here. The “intensive research design” is used in research where we wish to obtain in-depth knowledge of a specific phenomenon for the purpose of causal explanation. “Intensive research design” mainly applies to qualitative methods. “Extensive research” typically uses more quantitative methods that seek to identify regularities and patterns. The “extensive” study typically identifies regularities and has limited explanatory power (i.e. of how and why).

A realist approach to evaluation of programme mechanisms was advanced by Pawson and Tilley [34]. The approach assumes that whenever a programme is implemented it is testing an existing programme theory consisting of realist programme hypotheses (CMOs). This is assumed to be the case even if the theory was not made explicit. Consequently one of the tasks of a realist programme evaluation is to make the programme theory explicit by developing clear realist programme hypotheses about how, and for whom, programs might ‘work’. Subsequently the evaluation of those programme hypotheses can be done. The Realist [Programme] Evaluation approach usually starts with a programme that has been designed. The process of designing a programme intervention using realist causal theory is not well explicated.

### Theory driven design and evaluation

Theory-driven approaches to the design and evaluation of complex community initiatives are informed by the Aspen Institutes Theories of Change framework [47,48], and the Realist Evaluation approach. Astbury and Leeuw [49] observe that the methodology for constructing or reconstructing programme theory varies significantly. They observe that programme theory can be developed before a programme is implemented or after it has been running for some time. More often Theories of Change and realist evaluation start from the basis of an existing programme [34,48,50]. Consequently the translation of explanatory causal theory to design theory and programme theory is not well described. The use of realist synthesis goes some way to addressing this translation process but is only useful if there have been previous programmes of a similar nature.

For the purposes of this protocol we have drawn on the work of Keller and colleagues [51] who present a realist design-evaluation framework that combines design theory and realist evaluation. In their model programme, kernel [core] theories are used to develop the design propositions which are evaluated by realist evaluation, resulting in further refinement of the programme or kernel theories.

The Theory Driven Evaluation (TDE) approach is concerned with the evaluation of a programmes impact. As with realist evaluation the first task is to make the programme implementation theory explicate. Renger and colleagues [52] note that this is not required for other approaches to process evaluation but it is necessary when undertaking a theory driven approach as in our research protocol. They further argue that the “*articulation of [the] implementation theory is essential for a meaningful process evaluation to be completed*”.

Blamey and Mackenzie [30] examine these matters further in their comparison of Theories of Change and Realist Evaluation. Citing Weiss [53, p 58] they define “implementation theory” as “*what is required to translate objectives into ongoing service delivery and programme operation*” and “programme theory” as ‘*the responses of the people to programme activities*’. Blamey and Mackenzie [30] also observe that *programme theory* is referred to as ‘middle-range’ theory by Pawson and Tilley [34].

Blamey and Mackenzie [30], propose that Theories of Change be used as a means of explicating *implementation theory* for the purpose of programme planning, improvement and the development of robust monitoring systems at a macro programme level; while realist evaluation approaches be used to examine micro level aspects of the most promising *programme theories*.

Thus for the programme of research described here we will: 1) develop programme theory based on the causal theory and then apply that to deliberately designed interventions [46,51]; and 2) confirm the causal theory using methods proposed by Sayer [36, p 243].

### Research Design

The research settings will be in metropolitan Sydney. The research design will be a longitudinal, multi-level, mixed method realist evaluation of applied programme interventions that seek to break the intergeneration cycle of social disadvantage and poor child health and developmental outcomes. The programme interventions are likely to include: targeted home visiting and parenting services, place-based community and school initiatives, and whole of system “collective impact” and integrated care approaches.

In summary, programme theory will be developed using the causal theory developed as part of the *Emergent* and *Construction Phases* previously described, together with other relevant published theories, meta-syntheses and realist synthesis. The programme design propositions and hypotheses will be expressed, in realist terms, as context-intervention-mechanisms and outcome (CIMO) conjectures, which will thus render the full constituents of the programme theory. Intervention initiatives will be designed and implemented by interagency collaborations that draw from the local government, health, education and social care sectors. In doing so, we aim to move from explaining underlying social mechanisms to generate social interventions in partnership with the affected populations [29].

The intervention design process will use a theory driven approach utilizing Theory of Change (ToC) and related logic models [54]. Implementation theory will be developed, and the initial programme theory adapted for use in real world circumstances (i.e. concrete contexts). Using a longitudinal realist evaluation approach hypotheses will be refined through ongoing data collection and analysis.

The research programme will consist of three phases:

1. **Operationalisation** of the theory and design of programme initiatives for implementation
2. **Evaluation** of the translated programme and implementation theory using critical realist evaluation
3. **Theory Testing** of realist hypotheses using both intensive and extensive critical realist research methods.

Ethics approval to conduct this research has been sought and obtained from the Sydney and South Western Sydney Local Health District Ethics Committees.

### Operationalisation

#### Stage 1: Causal and Programme Theory

The purpose of the Operationalisation Phase is to move from abstract *causal* theories to concrete applied *implementation* and *programme* theory. In the operationalisation phase we will: expand the layered domains used for realist causal MCO hypotheses, and the number of MCO hypotheses; identify relevant programme theory from other relevant published theories, meta-syntheses and realist synthesis; map context-intervention-mechanism-outcome links, and generate suitable MCO and CIMO hypotheses for empirical testing and programme evaluation respectively.

##### Expand Layered Domains

We will expand the layered domains used for the MCO hypotheses and revisit the psychological and social layers, with a particular focus on mechanisms identified in the Stress Process Model [55]. The Stress Process Model is concerned with explaining ways in which social structures influence mental health with a focus on the connection between disadvantaged social status and psychopathology. The *Construction* phase of our previous explanatory theory building limited the development of realist causal hypotheses to the psychological-social layers related to maternal stress and depression [20]. Abductive and retroductive analysis undertaken during theory development, and theory construction also identified potential MCO configurations in other domains which will be relevant to programme development and evaluation. Those domains included, for example: access to services and information; strengthening of social capital, social cohesion and social inclusion; the role of local government, housing and social care services, media and corporate business (Figure 2). The intention will be to make explicit the *laminated system* in the manner as described by Bhaskar and Danermark [40]. The output of this analysis will be a table of causal MCO configurations for which appropriate intervention and programme theory will be sought.

##### Relevant Programme Theory

Using the expanded layered domains and causal MCO configurations as a framework, we will identify prospective intervention and programme theory from relevant published theories, meta-syntheses and realist synthesis. The methods used will include: a literature review of published theories, meta-syntheses and realist syntheses using search terms derived from the expanded domains and causal MCO configurations; and Delphi studies in areas judged to be critical to subsequent programme design. The table of causal MCO configurations will be modified to include: causal mechanisms (M_C_), prospective programme interventions (I), and programme mechanisms (M_P_) thus, developing design propositions following the context-intervention-mechanism-outcome (CIMO) logic (Denyer, [46]. The identified programme theory will be tested as part of the design and implementation of suitable initiatives.

#### Stage 2: Initiative Design

In the second stage of the Operationalisation Phase we will undertake: collaborative design of suitable initiatives using theory driven approaches; define the implementation theory; apply programme theory to the logic model; and outline an evaluation approach.

##### Collaborative Design

New South Wales (NSW), Australia introduced the *Families First* initiative in July 1999. The aim of *Families First* was to support families and communities to care for children. The initiative draws on existing services and resources, and had a strong focus on coordinating network of services. The initiative was later renamed *Families NSW* and had a foundation of local interagency groups supported by programme management groups (PMGs) at District level. Collaborative planning will be used to develop suitable initiatives that can be used to operationalise the programme theory.

The nature of those initiatives cannot be determined with certainty, but are likely to involve interagency approaches to prevention and early intervention that identifies and supports those children and families most likely to require further assistance. The interventions currently being considered include: perinatal coordination, home visiting, place-based initiatives, parenting programmes, and school transition initiatives.

Collaborative design will determine the approach which will be taken, but the use of theory-based evaluation methodology will be preferred. Sector training in Results Based Accountability [56], a data driven decision making process, has provided a foundation for collaborative theory informed planning of programmes. Consequently, it is envisaged that Theory of Change and Logic Models will be able to be constructed.

The difficulties of developing Theory of Change through collective and collaborative processes are, however, well recognised [57]. Mackenzie and Blamey outline a set of steps that if followed will result in identifying an initiatives Theory of Change. “Those steps are as follows:

1. Identification of the long-term outcomes that the initiative seeks to achieve
2. Identification of the interim outcomes and contextual features that will be required to meet these longer-term outcomes
3. Specification of the activities that will be put into place and the contextual requirements to realise those interim outcomes
4. An explicit recognition of the resources that will be required to turn those goals into reality” [57].

Using a critical realist approach, the collective design will also require a historical analysis to be undertaken to elaborate the pre-existing structures and mechanisms contributing to the observed maternal, child and family outcomes [58].

##### Define Implementation and Programme Theory

The theory-driven approaches will help in making clear the inputs, activities and outcomes expected. This is usually visually expressed as Logic Models or results chains. A key feature of theory driven evaluation is the need to know what components of the intervention contribute to achieving its impact. It is necessary to understand the theory that underpins the mechanisms and programme mediators. Intervention theory applies to the ‘nuts and bolts’ of the intervention (i.e. activities, target groups, settings) and programme theory relates to the ‘responses of the people to programme activities’ (i.e. psychological mechanisms) [59]. We will review the logic models or results chains developed as part of the collaborative Theory of Change process and add appropriate programme theory mechanisms. This process may require additional literature reviews to those undertaken in Stage 1 of the Operationalisation Phase.

### Evaluation

The evaluation phase will be longitudinal with ongoing data collection, and refinement and augmentation of the theory and hypotheses developed in the Operationalisation Phase by drawing on emerging empirical evidence. We will collect qualitative and quantitative data on the context, mechanisms, intervention implementation, receipt, reach, acceptability and normalization (i.e. likely sustainability). A focus of the data collection will be on how intervention mechanisms interact with causal mechanisms and pre-existing context to generate changes in outcomes (also referred to as demi-regularities).

#### Stage One: Contextualisation of Case Studies

Danermark [35, p 168] observed that in order to explain we must study how mechanisms manifest themselves in concrete contexts. The initiatives to be evaluated will be complex with likely multiple contexts and layers as described by Layder [41, p 73] above. We anticipate that it will be necessary to focus the evaluation on one level and stage of the logic model (i.e. case-studies). The description of the various contexts will require a full historical and current perspective of the layered context. At the individual client level the contextualization will entail a full personal and family history similar to that undertaken in a comprehensive social interview. Where the evaluation is focusing on a situated activity or setting, the documentation is likely to require an exploration for historical pre-existing features of the setting that may themselves be mechanisms with generative power. Given the nature of the causal theories being investigated we intend to, where possible, focus on: 1) maternal and family contexts; 2) practitioner contexts; 3) place-based settings; and 4) interagency contexts. The pre-existing vertical relationships in the laminated system will also be examined.

#### Stage Two: Concretisation and Instrumentation

The implication of the above is that the evaluation will examine causal and programme interventions in different concrete situations. The hypotheses developed in the Operationalisation Phase will be used to develop data collection tools and approaches for those ‘concrete situations’. It is likely that modifications will be required for interview, focus group, and quantitative instruments to ensure acceptability, appropriateness and validity. For the purposes of our programmes based in Sydney, modifications will be required for data collection from Aboriginal and Torres Strait Islander populations, and those of ethnic and culturally diverse backgrounds. It will also be necessary to modify our data collection approach where domestic violence and severe psychological or physical trauma has been experienced. Given the emergent longitudinal nature of the research we anticipate that the data collection tools will require modification after each analytical cycle.

#### Stage Three: Realist Evaluation Data Collection

A mixed method approach will be undertaken to data collection. We will take an integrated approach to methods, data collection and analysis [60,61,62]. Yin [60] argues that without such integration “*different methods may sit in parallel, potentially leading to multiple studies, and not the desired ‘mixing’ of methods implicit in mixed methods research*”. Yin proposes that integration should occur in relation to: research questions, units of analysis, samples for study, instrumentation and data collection methods, and analytic strategies. The research design will strive to achieve the standards of integration proposed by Yin with integration occurring through use of common research questions, study design, units of analysis, samples for study and analytic strategies during both emergent and construction phases. We will assess the quality of the mixed method approach using frameworks proposed by Teddlie and Tashakkori [63, p 300] and Onwuegbuzie and Johnson [64].

##### Qualitative Data

The nature of the interventions to be evaluated has not yet been determined. The qualitative methods will be tailored to each intervention, and specifically to the programme theories and contexts being studied. Realist methodology is permissive of the data collection methods used and can draw from the traditions of phenomenology, grounded theory, and interpretivism. Qualitative data will be collected, for example, from:

- Documentary sources
- Case-notes completed by the intervention deliverers (i.e. community workers, facilitators, educators, clinical staff)
- Researcher direct observations
- Interviews with intervention deliverers, interagency partners and consumers of the intervention
- In-depth case studies involving participant observation, focus groups and interviews in a selection of settings
- Delphi surveys
- Social network analysis studies.

The qualitative research will capture the participants own understandings and meanings of the intervention and what is working, for whom, in what context. The research may suggest hypotheses about the complex mechanisms by which the intervention may, or may not work. It can be anticipated that mechanisms will be identified that were under-theorised in Phase 1. As observed by Jamal and colleagues [65], the “*qualitative data may provide important insight into contexts and unintended pathways that can then be tested via quantitative mediation and moderation analyses*”. As argued elsewhere, we will therefore, use an emergent theory approach to refine the theory and CIMO hypotheses prior to the quantitative model testing.

##### Quantitative Data

Quantitative data will be used for both programme and implementation evaluation. Those two purposes are quiet distinct with the instruments chosen for programme evaluation being derived from both the causal (MCO) and programme (CIMO) hypotheses developed in Operationalisation Phase, and subsequently modified during the implementation evaluation. Given the longitudinal emergent nature of the evaluation it is anticipated that some quantitative measurements will be added or altered during the course of the evaluations. We consider that addition or amendment of quantitative measures enables more valid testing of the middle range theories to be undertaken in the Theory Testing Phase.

#### Stage Four: Intervention Evaluation Data Collection

It is anticipated that a number of the interventions will be subject to implementation process reporting requirements determined by external funders. For the process evaluation to be meaningful it is important to articulate the implementation theory as discussed above. While implementation theory pertains to programme activities, the intention is to capture not only the steps of an activity but also the essence of how the activities affect the mechanisms of change identified in the programme theory [52]. The intervention data collection will, therefore, focus on capturing two types of data: 1) whether the programmes were delivered as intended; and 2) the process of implementation and how the activities influenced the hypothesised programme interventions (CIMO).

#### Stage Five: Refining the Intervention

As stated above, the evaluation phase will be an emergent longitudinal study with ongoing data collection, and refinement and augmentation of the theory and hypotheses developed in Operationalisation Phase by drawing on emerging empirical evidence. Such an approach is widely used in realist evaluation and is in keeping with the realist evaluation cycle [34, p 85, 66, p 29]. The evaluation of programme theory will be used to inform the intervention design, and may in certain circumstances lead to modification of the actual implementation.

Evaluation of the Intervention process, implementation theory and programme theory will almost certainly result in modification to the actual intervention design and implementation. This “*action*” approach to intervention implementation is widely accepted within health and social care, where trial and learning methodology are increasingly used. The use of PDSA (plan, do, study, act) cycles [67] within NSW health sector evaluations is common and will be used where appropriate or required by funding agencies.

### Theory Testing and Triangulation

The theory testing phase will test hypotheses using quantitative and qualitative studies, and further refine causal, programme and implementation theory. The empirical analysis will include 1) intensive (qualitative) studies, case studies and extensive (quantitative) modelling to test causal CMO hypotheses arising from the *Operationalisation Phase*; and 2) quantitative modelling of CIMO hypotheses derived from the *Evaluation Phase*. Triangulation of the empirical, process and outcomes studies will be used to refine the causal, programme and implementation theories.

#### Realist evaluation of causal hypotheses

Separate from the evaluation studies described above we intend to undertake mixed method intensive studies, case studies and quantitative modelling to test and refine causal hypotheses arising from the Operationalisation Phase. The intensive studies will use interviews, focus groups and concept mapping methods. The critical realist case studies will use the approaches described by Sayer [36], Maxwell [68], Easton [69] and Yin [70]. The extensive (quantitative) modelling studies will use multi-level, spatial and structural equation modelling methods. The purpose of the empirical studies will be to 1) replicate and extend our earlier studies, and 2) study the causal theories in settings quite separate from the programme evaluation(s).

#### Realist modelling of programme mechanisms

We will use the structural modelling approach recently described by Jamal and colleagues [65]. In keeping with earlier realist studies by Kazi [66] the programme evaluations will use previously validated psychometric instruments as measures of hypothesised mechanisms and outcomes. For example measures of child development and behaviour, self-reported health, self-efficacy, depression, isolation, and health literacy.

## Discussion

We have described here a realist translational social epidemiology protocol for a programme of research that will use the meta-theory of critical realism to concretise and contextualise previously described critical realist theory of neighbourhood context, stress, depression and the developmental origins of health and disease. We will situate these studies in the socially disadvantaged regions of Sydney where the local child and family inter-agencies are collaborating to design and implement new programme interventions based on our earlier studies of perinatal, child, youth and family outcomes [71,72,73,74]. Of particular concern to our communities are the significant disparities in early childhood development and adolescent outcomes that might be attributed to neighbourhood circumstances, family stress and intergenerational cycles of disadvantage and poor mental health.

The application of realist methodologies to social epidemiology and population health interventions is relatively new. O’Campo and Dunn [38] have recently observed that “*if social epidemiology continues in its current path, we are likely to see a continued growth in empirical studies demonstrating the existence of a variety of different health inequalities, with relatively little contribution to studies that characterise and inform solutions to those inequalities*”. We contend that the identification of solutions requires that we change approach from identifying associations or regularities in empirical data to the identification of the causal explanatory mechanisms, and consequently the application of programme interventions that impact on those causal mechanisms. In undertaking a programme of explanatory theory building, we have responded to the call to contribute to social theory for informing translational social epidemiology. The planned realist multilevel mixed method studies will identify individual level explanatory mechanisms, and operationalised postulated social and cultural structures. The abductive and retroductive theory building constructed middle-range theories, which we propose will be formally tested through theory driven evaluation, realist programme evaluation, case studies and statistical modelling.

In preparing this methodology we transverse several areas of epistemological and methodological controversy including: critical *versus* scientific realism; MCO, CMO and CIMO forms of realist propositions; causal, programme and implementation theory; Theory of Change *versus* realist evaluation; and the methodological place of statistical structural modelling within a critical realist epistemology. Based on the philosophical analysis advanced by Maxwell [68] we find no justification to reject the application of critical realist ontological and epistemological meta-theory to programme evaluation. We also contend that the realist evaluation and realist synthesis methods advanced by Pawson and Tilley [33,34] are consistent with Maxwell’s position. Pratschke [75] refutes, successfully in our opinion, the scepticism of some critical realist philosophers toward statistical methods. His views are supported by Olsen [76,77,78,79], Mingers [80] and others. Latent variables and structural equation modelling (SEM) also has a strong realist foundation within the Latent Variable Theory [81]. Consequently SEM will have important utility in testing our critical realist causal and programme theories.

## Conclusion

Central to this methodology is the development of programme theory. Much of the theory driven and realist evaluation literature begins with existing interventions. The first task in those situations is to identify the underlying programme theory. In preparing this programme of work we were faced with the translation of causal theory to programme and implementation theory. We have proposed as a first step in this methodology the formal translation of the middle-range ‘causal’ theory into a middle-range ‘programme theory’ followed by an intervention design process based on Theory of Change approaches. The work of Denyer and co-authors [46] is helpful here in making explicit the requirement for a design step in the realist evaluation cycle and Keller and colleagues [51] introduce CIMO logic as useful step in the translation process.

It is important to acknowledge here the important contribution that shared visions and collective planning will make to the development of successful Theory of Change. Consumer and practitioner input to the design and evaluation of interventions is critical to their success. A challenge to critical realist practice is the presentation of complex abstract constructs in simple language. Particularly important will be the communication of causal hypotheses. As previously observed, the strength of the critical realist approach will be the extent to which this paradigm can support the epistemological, ontological, axiological, methodological and rhetorical positions of applied translational social epidemiological research in concrete contexts.
